# Technological properties and probiotic potential of *Lactobacillus fermentum* strains isolated from West African fermented millet dough

**DOI:** 10.1186/s12866-015-0602-6

**Published:** 2015-11-11

**Authors:** James Owusu-Kwarteng, Kwaku Tano-Debrah, Fortune Akabanda, Lene Jespersen

**Affiliations:** Department of Applied Biology, Faculty of Applied Sciences, University for Development Studies, P. O. Box 24, Navrongo Campus, Navrongo, Ghana; Department of Nutrition and Food Science, University of Ghana, P. O. Box 134, Legon-Accra, Ghana; Department of Food Science, University of Copenhagen, Rolighedsvej 26, DK 1958 Frederiksberg C, Denmark

**Keywords:** Starter culture, *Lactobacillus fermentum*, Cereal, Traditional fermentation, Probiotics traits

## Abstract

**Background:**

Throughout Africa, food fermentations are still driven by indigenous microorganisms which influence the nutritional, organoleptic and safety of the final products. However, for improved safety, consistent quality and beneficial health effects, a trend has emerged which involves the isolation of indigenous strains from traditional fermented products to be used as functional starter cultures. These functional starter cultures possess inherent functional characteristics and can contribute to food quality and safety by offering one or more organoleptic, nutritional, technological or health advantage (probiotics). With the aim of selecting potential probiotic starter cultures, *Lactobacillus fermentum* strains isolated from fermented millet dough were investigated for technological properties and probiotic traits in-vitro.

**Results:**

A total of 176 *L. fermentum* strains were assessed for technological properties including rate of acidification, exopolysaccharide production and amylase activity. Following this, 48 strains showing desirable technological properties were first screened for acid resistance. Sixteen acid resistant strains were assessed for additional probiotic properties including resistance to bile salts, bile salt hydrolysis, antimicrobial property, haemolysis and antibiotics resistance. *L. fermentum* strains clustered into 3 groups represented by 36 %, 47 % and 17 % as fast, medium and slow acidifiers respectively. About 8 %, 78 % and 14 % of the strains showed strong, weak and no exopolysaccharides production respectively. Amylase activity was generally weak or not detected. After exposure of 48 *L. fermentum* strains to pH 2.5 for 4 h, 16 strains were considered to be acid resistant. All 16 strains were resistant to bile salt. Four strains demonstrated bile salt hydrolysis. Antimicrobial activity was observed towards *Listeria monocytogenes* and *Staphylococcus aureus* but not *E. coli* and *Salmonella enteritidis. Lactobacillus fermentum* strains were generally susceptible to antibiotics except 6 strains which showed resistance towards streptomycin, gentamicin and kanamycin.

**Conclusion:**

In vitro determination of technological and probiotic properties have shown strain specific difference among *L. fermentum* strains isolated from fermented millet dough. Sixteen (16) *L. fermentum* strains have been shown to possess desirable technological and probiotic characteristics in vitro. These strains are therefore good candidates for further studies to elucidate their full potential and possible application as novel probiotic starter cultures.

## Background

The consumption of fermented foods contribute immensely to human diet in many countries around the world. Throughout Africa, food fermentations are still driven by indigenous microorganisms in the raw ingredients [1–3] which influence the nutritional availability, organoleptic quality and safety of the final products [4, 5]. However, for improved safety and the production of fermented foods with consistent quality and beneficial health effects, a trend has emerged which involves the isolation of wild-type strains from traditional fermented products to be used as functional starter cultures in food fermentation [6, 7]. These functional starter cultures are starters that possess inherent functional characteristics and can contribute to food quality and safety by offering one or more organoleptic, nutritional, technological or health advantage (probiotics) [8]. Thus, the implementation of carefully selected strains as starter cultures or co-cultures in fermentation processes can help to achieve in situ expression of the desired property, maintaining a perfectly natural product and still function as probiotics (impart health benefit unto the consumer) where applicable.

*Lactobacillus fermentum* has been identified as the predominant lactic acid bacteria (LAB) specie in several African cereal based fermented foods [1, 3, 9–12]. The predominance of *L. fermentum* during *koko* production, a millet-based fermented porridge in northern Ghana, was reported by Lei and Jakobsen [11] and the biodiversity of *L. fermentum* in their study was revealed by pulsed field gel electrophoresis (PFGE) and by multivariate data analysis. Similar results were demonstrated by randomly amplified polymorphic DNA (RAPD)-PCR fingerprinting patterns for fermented maize [9]. The technological roles of *L. fermentum* including acidification and aroma formation has also been described for Ghanaian fermented maize dough [13, 14]. Despite the significant importance of *L. fermentum* in food fermentation, strains of this species isolated from spontaneously fermented food products in Africa are still rarely dealt with in scientific publications and detailed examinations of their technological properties, their ability to survive the passage of the gastrointestinal tract as well as their susceptibility to common antibiotics are still missing.

In a framework to describe specific characteristics of *L. fermentum* strains isolated from African fermented cereals and to select and develop functional starter cultures with probiotic effect for the production of traditional fermented foods, predominant microorganisms associated with the traditional processing of fura, a millet based fermented food in Ghana were first isolated and identified [1, 2]. In this paper, *L. fermentum* strains originating from traditionally fermented millet dough were evaluated for their technological properties. Their ability to survive the passage of the gastro-intestinal tract, haemolytic activities, antimicrobial properties and susceptibility to several antibiotics were investigated. This is geared towards the selection and further development of probiotic starter cultures.

## Methods

### Bacterial strains

A total of 176 *L. fermentum* strains, isolated from spontaneously fermented millet dough were screened for some technological and probiotic properties, following a series of in vitro tests. The *L. fermentum* strains were previously isolated and identified by (GTG)_5_ – based rep-PCR fingerprinting and sequencing of their 16S rRNA [GenBank:JF268321 - JF268326], as described by [1].

Indicator strains for antimicrobial activity included *Escherichia coli* O157 882364, *Salmonella enteritidis* ATCC 13076, *Listeria monocytogenes* NCTC 10527 and *Staphylococcus aureus* ATCC 1448 which were cultured and maintained in Luria–Bertani (LB), Nutrient Broth (NB), Brain–Heart Infusion (BHI) and Tryptic Soy (TS) media respectively.

### Determination of technological properties

#### Acidification of millet broth

Fermentations trials were conducted by inoculating *L. fermentum* isolates into sterile millet broth and measuring the change in pH over time. For the preparation of millet broth, whole millet grains were cleaned by washing three times with distilled water. The washed grains were dried in an oven at 60 °C for 90 min and dry milled using a disc plate attrition mill (Hunt no. 2A & Co., Kent, UK). Millet broth was prepared as an aqueous suspension 10 % (w/v) in distilled water, dispensed into conical flasks (200 ml per flask) and autoclaved at 115 °C for 10 min. A loopful of an overnight culture was collected from MRS agar, transferred into 10 ml MRS broth and incubated at 30 °C for 24 h. About 100 μl of the 24 h old culture were transferred into 10 ml MRS broth and incubated at 30 °C for 16 h. Subsequently, cells were harvested by centrifugation at 5000 *g* for 10 min (4 °C), washed three times with 20 ml sterile diluent [0.1 % (w/v) peptone, 0.85 % (w/v) NaCl, pH 7.2 ± 0.2], and finally re-suspended in 10 ml of sterile diluent. This suspension served as the isolate inoculum and was sampled for viable cell count on MRS agar. Flasks containing 200 ml of autoclaved millet broth were inoculated in duplicates to obtain initial cell counts of *ca* 10^6^ cfu/ml, and incubated at 35 °C. About 200 ml of sterile millet broth served as a negative control. Samples were aseptically collected at 3 h intervals over 24 h period for measurement of pH.

#### Exopolysaccharide production

The screening of *L. fermentum* isolates for their ability to produce exopolysaccharide (EPS) was carried out as described by [15]. Bacterial cells from fresh overnight (18 h) cultures on MRS agar were streaked on LTV agar [0.5 g/l tryptone, 10 g/l meat extract, 6.5 g/l NaCl, 8 g/l potassium nitrate, 8 g/l sucrose, 0.1 % (v/v) Tween 80, 17 g/l agar, pH 7.1 ± 0.2] and incubated at 35 °C for 48 h. The stickiness of colonies were determined by the inoculating loop method [16]. Isolates were tentatively considered positive for exopolysaccharide if the length of slime was above 1.5 mm. Positive isolates were confirmed using MRS – sucrose broth without glucose and peptone as follows: [1 % (w/v) meat extract, 5 g/l yeast extract, 50 g/l sucrose, 2 g/l K_2_HPO_4_^.^3H_2_O, 5 g/l sodium acetate trihydrate, 2 g/l triammonium citrate anhydrous, 0.2 g/l MgSO_4_.7H_2_O, 0.05 g/l manganese (II) sulphate monohydrate, 0.1 % (v/v) Tween 80, pH 5.0 ± 0.2]. The isolates were then incubated at 30 °C for 24 h. A volume of 1.5 ml of the 24 h culture was centrifuged at 5000 *g* for 10 min (4 °C). About 1 ml of the supernatant was put in a glass tube and an equal volume of ethanol (99 %) was added. In the presence of EPSs, an opaque link is formed at the interface.

#### Amylase activities

The ability of *L. fermentum* to produce amylase was determined according to the method described by [17]. Active cultures of LAB isolates were point-inoculated on modified MRS agar without glucose but with potato soluble starch as the sole carbon source. The media composition was as follows: [10 g/l tryptone, 10 g/l meat extract, 5 g/l (w/v) yeast extract, 20 g/l potato-soluble starch, 2 g/l K_2_HPO_4_.3H_2_O, 5 g/l sodium acetate, 2 g/l triammonium citrate, 0.2 g/l MgSO_4_.7H_2_O, 0.05 g/l manganese (II) sulphate monohydrate, 0.1 % (v/v) Tween 80, pH 5.0 ± 0.2]. Inoculated plates were incubated anaerobically (AnaeroGen, oxoid) at 35 °C for 48 h. The culture plates were covered by spraying with Lugol’s iodine [0.33 % (w/v) iodine, 0.66 % (w/v) potassium iodide] to detect starch hydrolysis. Un-degraded starch stains blue-black while the presence of a clear halo zone around a tested colony was taken as indication of starch degradation and therefore the production of α-amylase. Diameters of the halos around colonies were measured.

### Determination of probiotic properties

#### Resistance to low pH

Resistance to low pH was determined according to [18] and [19]. Bacterial cells from fresh overnight (18 h) cultures were harvested (10,000 x g, 5 min, 4 °C), washed twice with PBS buffer (pH 7.2), re-suspended (2 %) in PBS solution and adjusted to pH 2.5. Resistance was assessed in triplicates in terms of viable colony counts and enumerated on MRS agar (Merck) after incubation at 37 °C for 4 h, reflecting the possible time spent by food in the stomach.

#### Resistance to bile salts and bile salt hydrolysis

Bacterial cells from overnight (18 h) cultures were harvested (10,000 x g, 5 min, 4 °C), washed twice with PBS buffer (pH 7.2), before inoculating in PBS solution (pH 8.0), containing 0.3 %, 0.5 %, 1 % and 2 % (w/v) bile salt (Oxgall, Difco). Resistance was assessed in triplicates in terms of viable colony counts and enumerated after incubation at 37 °C for 4 h.

For the determination of bile salt hydrolysis (BSH), fresh bacterial cultures were streaked in triplicates on MRS agar containing 0.5 % (w/v) taurodeoxycholic acid (Sigma). The hydrolysis effect was indicated by different colony morphology from the control MRS plates, after 48 h of anaerobic incubation at 37 °C.

#### Estimation of survival rates

Survival rates for *L. fermentum* strains were estimated after their growth in low pH (pH 2.5) and different bile salt concentrations (0.3 %, 0.5 %, 1 % and 2 %).

#### Haemolytic activity

Fresh bacterial cultures were streaked in triplicates on Columbia agar plates, containing 5 % (w/v) human blood (Michopoulos S.A., Athens, Greece), and incubated at 37 °C for 48 h under anaerobic condition. Blood agar plates were examined for signs of β-haemolysis (clear zones around colonies), α-haemolysis (green-hued zones around colonies) or γ-haemolysis (no zones around colonies).

#### Antimicrobial activity

Fresh overnight *L. fermentum* culture supernatants were collected by centrifugation (10,000 x g, 15 min, 4 °C), adjusted to pH 6.5 and filter-sterilised (0.20 μm). The cell-free culture supernatants (CFCS) of the potential probiotic strains were screened for inhibitory activity against indicator pathogens (described in section 2.1) using the agar well diffusion method. Briefly, an initial inoculum of approximately 10^6^ cfu/ml of the target strain was incorporated into soft agar (1 %, w/v) plates of the appropriate medium for the indicator strain. CFCS (50 mL) were transferred into wells (5 mm diameter) drilled into the agar. The plates were incubated at 37 °C for 24 h, and the antimicrobial activity was recorded as inhibition zones (diameter) around the well. Kanamycin (30 mg/ml) was used as positive control, while MRS broth, adjusted to pH 6.5 and filtered served as the negative control.

#### Antibiotic resistance

For antibiotic resistance test, *L. fermentum* strains were inoculated (1 % v/v) in MRS broth supplemented with 9 different antibiotics (Ampicillin, Chloramphenicol, Tetracycline, Erythromycin, Streptomycin, Kanamycin, Gentamycin, Quinupristin/Dalfopristin, Clindamycin) at various final concentrations (1, 2, 4, 8, 16, 32, 64, 128, 256, 512, and 1024 μg/ml) and examined in triplicate for growth in a microplate reader (OD at 610 nm) following a 24 h incubation period at 35 °C.

## Results and discussion

### Acidification properties in millet broth

**Fig. 1:**
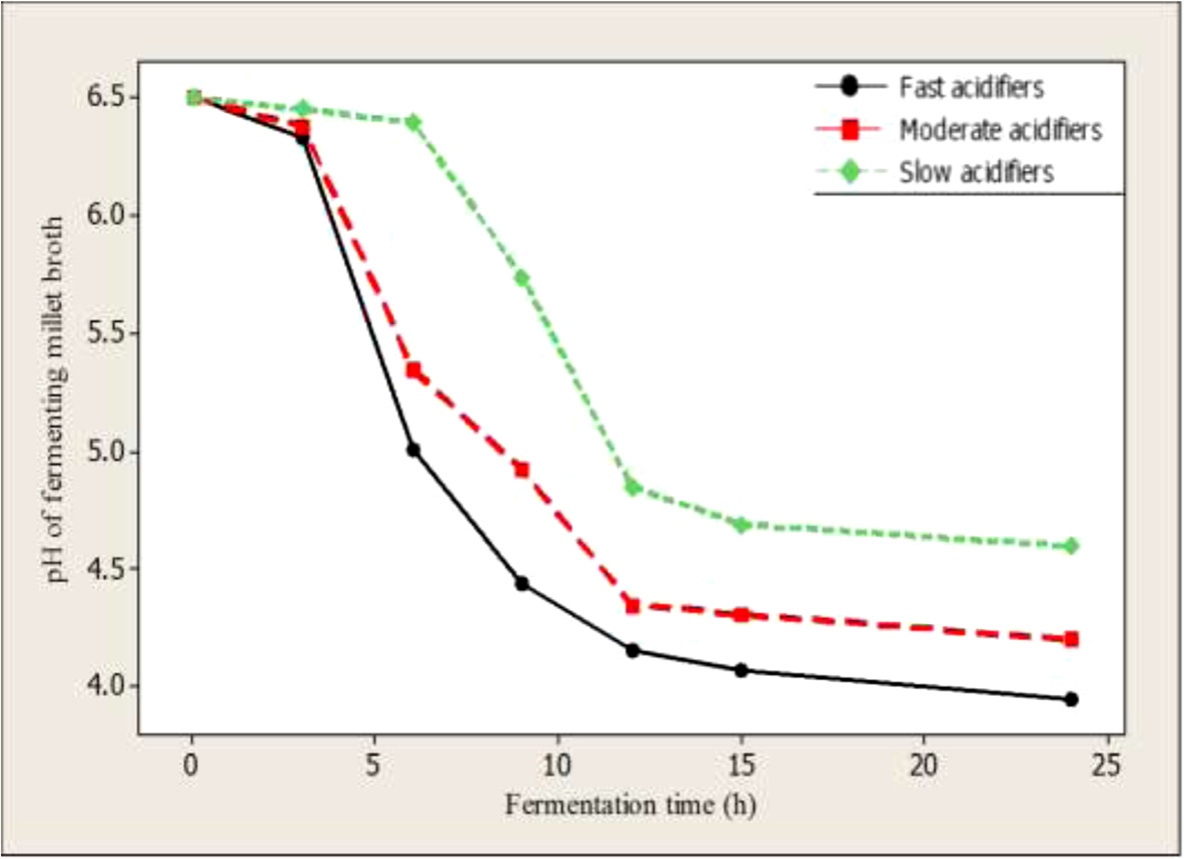
Rate of acidification by *L. fermentum* strains isolated from traditionally fermented millet dough. Values represent means ± standard errors of two independent experiments carried out in triplicate. The Tukey-Kramer test was used for comparison of means. Means with different capital alphabets are significantly different (*P* < 0.05) for each time point

*Lactobacillus fermentum* strains were clustered into three groups according to their acidification properties (Fig. 1). *Lactobacillus fermentum* isolates representing 36 % (fast acidifiers) and 47 % (moderate acidifiers) were able to obtain a change in pH (ΔpH) of 2 units after 9 h and 12 h of fermentation respectively. However, a third group of *L. fermentum* strains (slow acidifiers) representing 17 % of the total *L. fermentum* strains never attained pH change up to 2 pH units. The demonstration of faster acidification property by *L. fermentum* strains is a required technological property for the development of starter cultures for controlled fermentation processes as faster acidification is necessary for reducing fermentation time and reducing contamination by spoilage and/or pathogenic microorganisms.

### Exopolysaccharide production and amylase activity

Amylase activity and exopolysaccharides production by *L. fermentum* isolated from fermented millet are shown in Table 1. Amylase activity of the *L. fermentum* strains were generally weak or not detected. About 16.5 % of the total strains only showed weak amylolytic activity. Out of a total of 176 strains of *L. fermentum*, about 85.6 % showed slime formation while 14.4 % showed no slime formation or exopolysaccharides production.

**Table 1:** Amylase activity and exopolysaccharides production by *L. fermentum*

		Clear zone around colonies/slime length^a^			
Microbial specie	Activity	-	+	++	+++
*L. fermentum* (*n* = 176)	Amylase	83.5	16.5	0	0
	Exopolysaccharide	14.4	38.4	39.2	8.4

^a^Values are percentages (%) of the total number (n) of *L. fermentum* strains

- No clear zone around colony or slime formation observed, + diameter of clear zone or slime length of <1.5 mm, ++ diameter of clear zone or slime length of 1.5 – 3 mm, +++ diameter of clear zone or slime length >3 mm

Generally, high prevalence of amylase producing LAB has not been reported. However, few strains of *L. fermentum* isolated from fermented maize products have been reported as amylase producers [17, 20]. Amylolytic lactic acid bacteria from traditional fermented foods could be of economic interest in the production of lactic acid from direct fermentation of starchy products [21, 22]. Additionally, they may present the potential for decreasing the viscosity of bulky, starchy, weaning porridges which may enable an improvement in their nutrient density while maintaining an acceptable thickness for feeding young children in developing countries [23].

The ability of *L. fermentum* strains to produce EPS is not surprising since previous studies have shown that many food grade microorganisms produce EPS [12, 24]. Technologically, the physicochemical properties of EPS, such as viscosity, have motivated their utilization in food applications as, for example, biothickeners [24, 25]. Therefore, texture which is an important attribute associated with the consumption of traditional fermented cereal products will be affected by EPS produced by the selected *L. fermentum* starter cultures. Interestingly, The *L. fermentum* strains which produced EPS were found to be resistant to low pH condition (Table 5). The production of EPS has been reported to protect the producing microorganisms against dehydration and other harsh conditions such as acid and bile [26, 27], and may also contribute to the aggregation properties required for colonisation by probiotic lactic acid bacteria [28, 29].

### Resistance to low pH

Following a determination of technological properties, 48 *L. fermentum* strains were further assessed for resistance to low pH (2.5) in PBS over 4 h duration (Table 2). Using a survival rate of ≥ 80 % after incubation at pH 2.5 for 4 h, 16 *L. fermentum* strains were considered to be resistant to low pH 2.5 and were selected for further screening. This results confirm the strain-specific differences which exist among lactobacilli in relation to their probiotic properties. Similar results were obtained from previous reports, where *Lactobacillus* strains of food, human or animal origin, were able to retain their viability when exposed to low pH values between 2.5 and 4.0 [18, 30–34]. The pH value in human stomach may ranges from 1.5, during fasting, to 4.5, after a meal, and food ingestion can take up to 3 h [35]. The pH value (2.5) and 4 h duration used in this study for the selection of potential probiotic *L. fermentum* strains is very selective and even though it is not the most common condition in the human stomach, it assures the selection of the very acid-tolerant strains [36].

**Table 2:** Acid resistance of 48 *L. fermentum* strains grown in PBS at pH 2.5 for 4 h

Strains	Viable count (log cfu/ml)^a^		Survival rate (%)^b^
	0 h	4 h	
***L. fermentum*** **6–6**	**8.87 ± 0.02**	**7.36 ± 0.11**	**83.0**
***L. fermentum*** **f-7**	**9.11 ± 0.06**	**8.05 ± 0.04**	**88.4**
*L. fermentum* f-17	9.05 ± 0.10	6.48 ± 0.12	71.6
*L. fermentum* 6–1	8.70 ± 0.05	4.33 ± 0.03	49.8
*L. fermentum* 6–2	9.21 ± 0.05	6.00 ± 0.08	65.1
*L. fermentum* 10–4	9.03 ± 0.13	3.58 ± 0.05	39.6
***L. fermentum*** **10–9**	**9.45 ± 0.04**	**8.20 ± 0.00**	**86.8**
*L. fermentum* f-26	9.20 ± 0.06	7.04 ± 0.02	76.5
***L. fermentum*** **f-29**	**9.18 ± 0.03**	**8.10 ± 0.06**	**88.2**
*L. fermentum* 10–1	8.84 ± 0.11	3.65 ± 0.09	41.3
*L. fermentum* 12–5	8.92 ± 0.08	5.54 ± 0.05	62.1
*L. fermentum* 10–3	9.00 ± 0.01	6.35 ± 0.05	70.6
*L. fermentum* 4–12	8.96 ± 0.15	4.08 ± 0.10	45.5
***L. fermentum*** **12-18A**	**9.10 ± 0.07**	**8.26 ± 0.06**	**90.8**
***L. fermentum*** **12-19A**	**9.37 ± 0.05**	**7.99 ± 0.12**	**85.3**
***L. fermentum*** **12-20A**	**9.05 ± 0.08**	**8.35 ± 0.03**	**92.3**
*L. fermentum* 12-6A	9.38 ± 0.15	4.82 ± 0.10	51.4
*L. fermentum* 8-16A	9.33 ± 0.06	3.50 ± 0.08	37.5
***L. fermentum*** **4–20**	**8.66 ± 0.02**	**7.58 ± 0.10**	**87.5**
*L. fermentum* 4-12A	9.35 ± 0.05	4.65 ± 0.06	49.7
*L. fermentum* f-11A	9.22 ± 0.08	4.05 ± 0.12	45.0
*L. fermentum* f-22	9.40 ± 0.04	5.50 ± 0.05	58.5
*L. fermentum* f-2A	8.78 ± 0.05	4.80 ± 0.10	54.1
***L. fermentum*** **2–15**	**9.08 ± 0.10**	**8.20 ± 0.14**	**90.3**
*L. fermentum* 2-3A	9.08 ± 0.07	6.18 ± 0.04	68.0
*L. fermentum* 6-13A	9.20 ± 0.15	3.95 ± 0.10	42.9
***L. fermentum*** **0–17**	**9.35 ± 0.06**	**8.00 ± 0.07**	**85.6**
*L. fermentum* 2-23A	9.28 ± 0.10	3.20 ± 0.14	34.5
***L. fermentum*** **8–10**	**9.64 ± 0.10**	**7.95 ± 0.08**	**82.5**
***L. fermentum*** **0-25A**	**9.50 ± 0.04**	**8.06 ± 0.15**	**84.8**
*L. fermentum* 10–31	9.00 ± 0.06	6.50 ± 0.04	72.2
*L. fermentum* 2-14A	9.47 ± 0.12	4.45 ± 0.10	46.9
***L. fermentum*** **f-5A**	**8.85 ± 0.07**	**8.11 ± 0.05**	**91.6**
*L. fermentum* f-22A	8.91 ± 0.05	3.80 ± 0.12	42.6
*L. fermentum* 12–15	8.80 ± 0.15	5.65 ± 0.06	64.2
*L. fermentum* 10-19A	9.06 ± 0.07	3.90 ± 0.10	43.0
*L. fermentum* 10–24	7.98 ± 0.10	4.00 ± 0.08	50.1
*L. fermentum* 10-16A	9.18 ± 0.08	5.45 ± 0.15	59.4
*L. fermentum* 8–13	9.50 ± 0.14	4.85 ± 0.05	51.1
***L. fermentum*** **8-5A**	**9.06 ± 0.06**	**7.30 ± 0.10**	**80.6**
*L. fermentum* 8–20	9.35 ± 0.08	6.03 ± 0.07	64.5
***L. fermentum*** **8–28**	**9.28 ± 0.12**	**8.45 ± 0.09**	**91.1**
*L. fermentum* 6-18A	9.09 ± 0.10	6.60 ± 0.10	72.6
*L. fermentum* 6–23	9.11 ± 0.06	5.84 ± 0.05	64.1
***L. fermentum*** **4–30**	**9.36 ± 0.08**	**8.00 ± 0.16**	**85.5**
*L. fermentum* 4-16A	9.20 ± 0.14	5.36 ± 0.09	58.3
*L. fermentum* 4-10A	8.97 ± 0.05	2.66 ± 0.15	29.7
*L. fermentum* 12–7	9.06 ± 0.10	3.50 ± 0.07	38.6

^a^Values are means ± standard deviation of two independent experiments

^b^acid resistant strains with mean survival rates ≥80 % after 4 h in are in bold

In order for probiotic bacteria to fulfil their physiological role in the gut, the bacteria must overcome a number of stresses before they reach the target site [37]. The acidic environments encountered both in food and in the gastrointestinal tract provide a significant survival challenge for probiotic organisms. For example, the preferred delivery vehicles for probiotic cultures are acid fermented food products which present an acid challenge. In a situation where the probiotic bacteria is used as a starter culture for the fermentation, the potential probiotic organism require mechanisms to survive the adverse effects of the by-products (organic acids) of their own metabolism. In addition to their ability to survive the harsh environments encountered during processing, the bacteria will need to survive the highly acidic gastric juice if they are to reach the small intestine in a viable state [38]. Passage of probiotics through the gastrointestinal tract (GIT) is a stressful journey, with stress stages which may affect cell viability. The principal stress is that of shifting pH encountered in the stomach, resulting from gastric acid as well as bile [39, 40]. Hence, acid tolerance is accepted as one of the desirable properties used to select potentially probiotic strains.

### Resistant to bile and bile salts hydrolysis

**Fig. 2:**
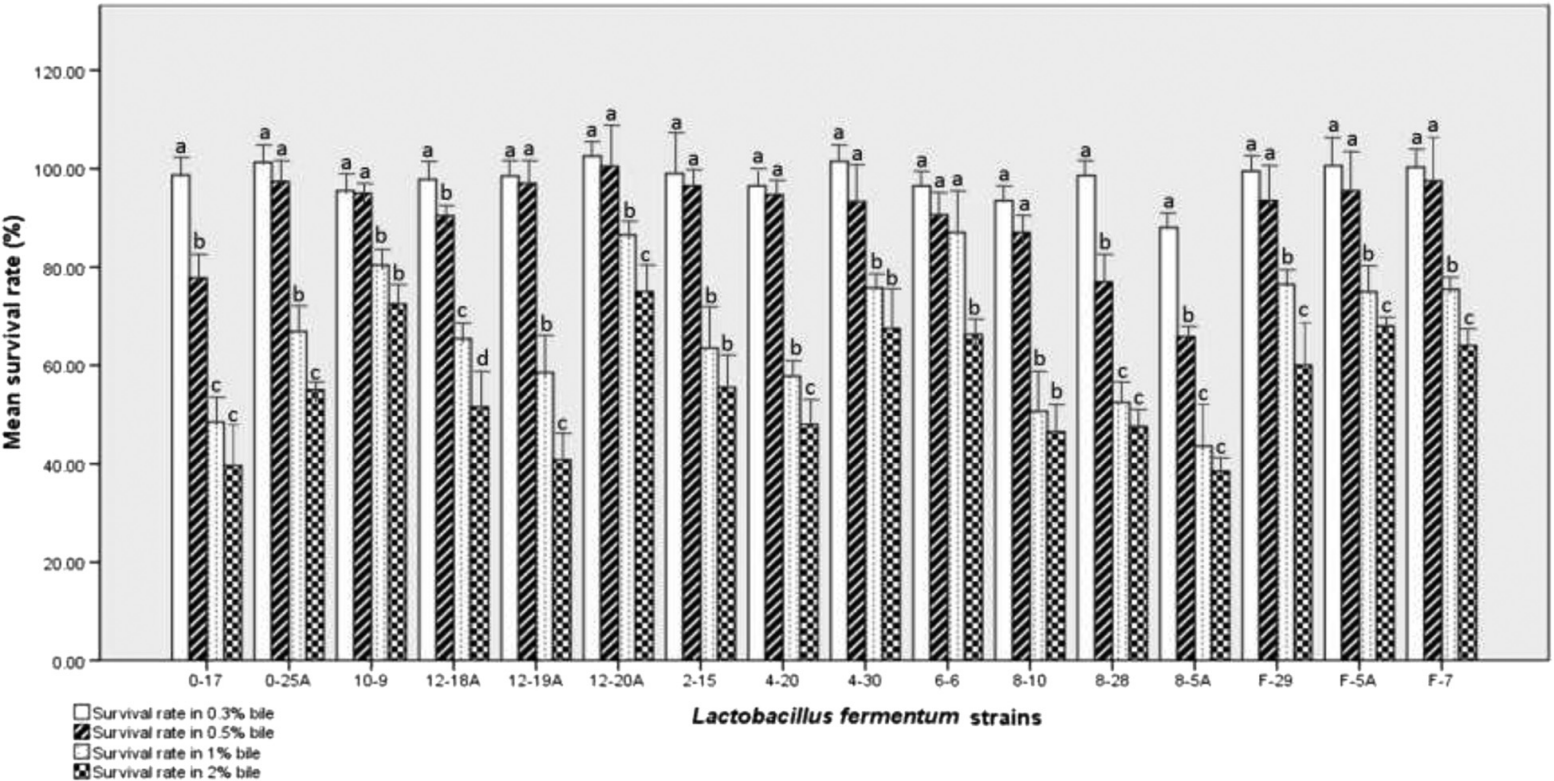
Survival rate (%) of *L. fermentum* strains after 4 h incubation in different bile salt concentrations. Values are given as the mean ± standard error of two independent experiments carried out in triplicate. The Tukey-Kramer test was used for comparison of means. Means with different alphabets within a strain are significantly different (*P* < 0.05)

The ability of *L. fermentum* strains to tolerate the effect of different concentrations of bile salt after incubation for 4 h is shown in Fig. 2. All 16 tested *L. fermentum* showed resistance (survival rate ≥ 80 %) to 0.3 % bile salt. However, resistance decreased significantly (P < 0.05) with increasing bile salt concentration.

The detergent property of bile confers potent toxicity, primarily through the dissolution of bacterial membranes [41]. Therefore, for a probiotic strain to be able to perform effectively in the gastrointestinal tract, it must overcome the antimicrobial challenge posed by bile. Thus in vitro resistance to bile has become necessary in screening potential probiotic strains as one of the physiologically relevant stresses in the gastrointestinal tract [33, 42].

While all *L. fermentum* strains in this study were able to grow in the presence of bile salt, only four (4) strains demonstrated the ability to hydrolyse taurodeoxycholic acid (TDCA), as indication of in vitro bile salt hydrolyse (BSH) activity. Some authors have suggested that BSH activity and resistance to toxicity of conjugated bile salts are unrelated properties in lactobacilli [43, 44]. The ability of probiotic strains to hydrolyse bile salts has often been included among the criteria for probiotic strain selection [45], although there are divided views on whether microbial bile salt hydrolase (BSH) activity is a desirable trait for probiotic strains. On one hand, blood cholesterol lowering effect has been correlated to the bile salt hydrolase activity of some lactobacilli [45, 46]. On the other hand, unconjugated bile acids are less efficient molecules in the emulsification of dietary lipids and so BHS may compromise normal lipid digestions, and subsequently, the absorption of fatty acids and monoglycerides could be impaired [47]. In general however, there is sufficient data to suggest that microbial BSH-activity function in the detoxification of bile salts increases the intestinal survival and persistence of producing strains, which in turn increases the overall beneficial effects associated with a probiotic strain [41, 45].

### Antimicrobial activity

None of the cell free neutralized supernatant (pH 6.5) showed antimicrobial activities against the pathogenic strains *Escherichia coli* O157 882364 and *Salmonella enteritidis* ATCC 13076. However, four *L. fermentum* strains (i.e. 10–9, 4–20, 0–17 and 4–30) showed inhibitions towards *Listeria monocytogenes* NCTC 10527 and *Staphylococcus aureus* ATCC 1448 (Table 3.). Previous reports on probiotic *L. fermentum* strains showed negligible antimicrobial activity in neutral pH 7.0 [48]. Moreover, it has been reported that neutralization of the soluble fraction to pH 6.5 significantly reduced the antimicrobial activity against pathogens [49].

**Table 3:** Antagonistic activity of 16 *L. fermentum* strains against selected pathogens

Bacteria strain	^a^Antagonistic activity	
	*L. monocytogenes* NCTC 10527	*Sta. aureus* ATCC 1448
*L. fermentum* 6–6	-	-
*L. fermentum* f-7	-	-
*L. fermentum* 10–9	++	++
*L. fermentum* f-29	-	-
*L. fermentum* 12-18A	-	-
*L. fermentum* 12-19A	-	-
*L. fermentum* 12-20A	-	-
*L. fermentum* 4–20	+	++
*L. fermentum* 2–15	-	-
*L. fermentum* 0–17	++	++
*L. fermentum* 8–10	-	-
*L. fermentum* 0-25A	-	-
*L. fermentum* f-5A	-	-
*L. fermentum* 8-5A	-	-
*L. fermentum* 8–28	-	-
*L. fermentum* 4–30	+	++

^a^Antagonistic activity was measure as diameter of inhibition zone as follows: (−) = < 1 mm, (+) = 1-2 mm, (++) = 3-4 mm, (+++) = > 4 mm. No antagonistic activity was observed towards others strains *E. coli* O157 882364 and *S. enteritidis* ATCC 13076

The prevention of gastrointestinal tract colonization by a variety of pathogens is a primary mechanism of beneficial effects mediated by probiotics [50]. The mechanisms underlying the antimicrobial activity of lactobacilli are believed to involve the production of different kinds of inhibitory substances and competitive exclusion [51]. The capacity to produce different antimicrobial compounds may be one of the critical characteristics for effective competitive exclusion of pathogen survival in the intestine and expression of a probiotic effect for the host [52]. The acidic conditions in the stomach may also enhance the activity of these antimicrobial compounds [53]. Furthermore, these probiotic characteristics may partly be based on the production of relevant concentrations of lactic acid in the microenvironment, which, in combination with a detergent such as bile salts, inhibits the growth of Gram-negative pathogenic bacteria [41]. However, antimicrobial mechanisms other than those driven by bacterial metabolites may play a significant role the antimicrobial property of probiotic in vivo.

### Haemolytic activity

None of the tested *L. fermentum* strains showed β-haemolytic activity. However, *L. fermentum* 0-25A and *L. fermentum* 12-18A showed α-haemolytic activity. Thus, almost all *L. fermentum* strains isolated from fermented millet dough expressed γ-haemolysis (i.e. no haemolysis). Similar observations were made for *Lactobacillus* spp. isolated from dairy products [18], fermented olives [30] and different African fermented food products [54]. The general absence of haemolysis or poor haemolytic activities expressed by lactic acid bacteria is indicative of their safety applications in food. In vitro assessment of haemolytic activity on blood agar is one of the safety requirements often used to assess potential probiotic strains [55]. On blood agar plates, microbial strains with β-haemolytic activity produce exotoxins which causes the lysis of blood cells, resulting in clearing of the zones around bacteria colonies.

### Antibiotic resistance

Minimum inhibitory concentration (MIC) of 9 antibiotics determined for 16 *L. fermentum* strains is shown in Table 4. Strains were considered resistant when they showed MIC values higher than the MIC breakpoints established by the European Food Safety Authority [56]. The majority of *L. fermentum* (*n* = 10) revealed low MICs for all tested antibiotics and are considered as non-resistant according to the EFSA breakpoint [56]. However, 6 strains showed resistance towards the protein synthesis inhibitor antibiotics streptomycin, kanamycin and gentamicin, mostly at low levels (MICs one to two log cycles above the cut-off point). [57] and [54] similarly reported gentamicin resistance in *L. fermentum* strains from African fermented foods.

**Table 4:** Minimum inhibitory concentrations of 9 antibiotics for 16 *L. fermentum* strains isolated from traditional millet fermentation

Bacteria strain	^a^MIC (μg/ml)								
	A	C	T	F	S	K	G	Q/D	CL
*L. fermentum* 6–6	<1	2	2	<1	8	16	8	<1	<1
*L. fermentum* f-7	1	4	4	<1	32	32	4	<1	<1
*L. fermentum* 10–9	1	4	2	1	128^b^	32	64^b^	1	<1
*L. fermentum* f-29	1	2	2	<1	4	64^b^	32^b^	<1	<1
*L. fermentum* 12-18A	1	1	2	<1	8	8	16	<1	1
*L. fermentum* 12-19A	<1	2	2	<1	128^b^	128^b^	32^b^	<1	<1
*L. fermentum* 12-20A	<1	2	8	<1	8	32	16	<1	1
*L. fermentum* 4–20	1	2	2	<1	8	16	16	<1	1
*L. fermentum* 2–15	1	1	2	<1	32	32	8	<1	<1
*L. fermentum* 0–17	1	1	4	<1	16	128^b^	16	<1	<1
*L. fermentum* 8–10	1	1	2	1	128^b^	64^b^	64^b^	2	<1
*L. fermentum* 0-25A	1	<1	1	1	64	32	8	<1	<1
*L. fermentum* f-5A	1	<1	1	<1	8	16	8	<1	<1
*L. fermentum* 8-5A	<1	2	4	<1	8	16	16	1	<1
*L. fermentum* 8–28	1	2	2	<1	128^b^	64^b^	32^b^	1	<1
*L. fermentum* 4–30	1	1	2	1	64	8	16	<1	<1

^a^ *MIC* Minimum inhibitory concentration

^b^Resistant according to the EFSA’s breakpoints (EFSA, 2008)

*A* ampicillin, *C* chloramphenicol, *T* tetracycline, *E* erythromycin, *S* streptomycin, *K* kanamycin, *G* gentamycin, *Q/D* quinupristin/dalfopristin, *CL* clindamycin

Safety concerns regarding the use of probiotics containing antibiotic resistant strains arise due to the possibility of transferring antibiotic resistant genes to intestinal pathogens [58]. However, according to previous studies [59–61] the antibiotic resistance observed for *Lactobacillus* strains are considered to be intrinsic or natural resistance because it is chromosomally encoded and, therefore, non-transmissible. Resistance to aminoglycoside antibiotics, such as gentamicin, streptomycin, kanamycin, is considered to be intrinsic in the Lactobacillus genus and is attributed to the absence of cytochrome-mediated electron transport, which mediates drug uptake [60, 62].

## Conclusion

In conclusion, in vitro determination of technological and probiotic properties have shown strain specific difference among *L. fermentum* strains isolated from fermented millet dough. Sixteen (16) *L. fermentum* strains have been shown to possess desirable technological and probiotic characteristics in vitro as summarised in Table 5. These strains are therefore good candidates for further studies to elucidate their full potential and possible application as novel probiotic starter cultures.

**Table 5:** Summary of the characteristics of 16 *L. fermentum* strains with technological and probiotic potential according to in vitro tests

Bacteria strain	Technological properties			Probiotic properties				
	^a^RA	^b^EPs	^c^AA	^d^AR (SR %)	^e^RBS (SR %)	^g^BSHA	^i^HA	^j^RAB
*L. fermentum* 6–6	F	H	-	83.0	96.5	0	γ	-
*L. fermentum* f-7	F	H	-	88.4	100.3	0	γ	-
*L. fermentum* 10–9	F	H	+	86.8	95.5	1	γ	S, G
*L. fermentum* f-29	F	H	-	88.2	99.5	1	γ	K, G
*L. fermentum* 12-18A	F	H	-	90.8	97.8	1	α	-
*L. fermentum* 12-19A	F	H	-	85.3	98.5	0	γ	S, K, G
*L. fermentum* 12-20A	F	H	+	92.3	102.6	0	γ	-
*L. fermentum* 4–20	F	H	+	87.5	96.5	0	γ	-
*L. fermentum* 2–15	F	H	-	90.3	99.0	0	γ	-
*L. fermentum* 0–17	F	H	-	85.6	98.7	1	γ	K
*L. fermentum* 8–10	F	H	+	82.5	93.5	0	γ	S, K, G
*L. fermentum* 0-25A	F	H	-	84.8	101.3	0	α	-
*L. fermentum* f-5A	F	H	-	91.6	100.6	0	γ	-
*L. fermentum* 8-5A	F	H	-	80.6	88.0	0	γ	-
*L. fermentum* 8–28	F	H	-	91.1	98.6	0	γ	S, K, G
*L. fermentum* 4–30	F	H	-	85.5	101.5	0	γ	-

^a^ *RA* rate of acidification, *F* fast acidifier

^b^ *EPs* Exopolysaccharides production, *H* high potential EPs producer

^c^ *AA* amylase activity, − no amylase activity, + weak amylase activity

^d^ *AR* acid resistance measured as mean survival rate (%)

^e^ *RBS* resistance to 0.3 % bile measured as mean survival rate (%)

^g^ *BSHA* bile salt hydrolase activity, *0* no hydrolysis, *1* partial hydrolysis

^i^ *HA* haemolytic activity, γ-haemolysis, α-haemolysis

^j^ *RAB* resistance to antibiotics, *S* streptomycin, *K* kanamycin, *G* gentamycin

## Abbreviations

ATCC: American type culture collection
BHI: brain–heart infusion
BSH: bile salt hydrolysis
CFCS: cell-free culture supernatants
DNA: de-oxyribonucleic acid
EFSA: European food safety authority
EPS: exopolysaccharides
GIT: gastrointestinal tract
LAB: lactic acid bacteria
LB: Luria–Bertani
MIC: minimum inhibitory concentration
MRS: de Man, Rogosa and Sharpe
NB: nutrient broth
NCTC: National collection of type cultures
PBS: phosphate-buffered saline
PCR: polymerase chain reaction
PFGE: pulsed field gel electrophoresis
RAPD-DNA: randomly amplified polymorphic- de-oxyribonucleic acid
rRNA: ribosomal ribonucleic acid
TDCA: taurodeoxycholic acid
TS: tryptic soy
